# Microneedle patch for immunization of immunocompromised hosts

**DOI:** 10.18632/oncotarget.22072

**Published:** 2017-10-25

**Authors:** Nadine G. Rouphael, Mark J. Mulligan

**Affiliations:** Nadine G. Rouphael: Department of Medicine, Hope Clinic of the Emory Vaccine Center, Division of Infectious Diseases, School of Medicine, Emory University, Atlanta, GA, USA

**Keywords:** microneedle patch, influenza, vaccine, self-administration, cancer

Influenza virus is a major cause of morbidity and mortality particularly in the immunocompromised host. When compared to the general population, influenza-related hospitalization rates are four times higher and mortality ten times higher in patients with cancer [1]. The Infectious Diseases Society of America (IDSA) recommends the administration of inactivated influenza vaccine (IIV) annually to patients with hematological or solid tumor malignancies except in patients receiving anti-B-cell antibodies or intensive chemotherapy [2]. Despite this recommendation, vaccine coverage in patients with malignant disease is quite low (18%)[3] and the vaccine response is poor.

**Figure 1:**
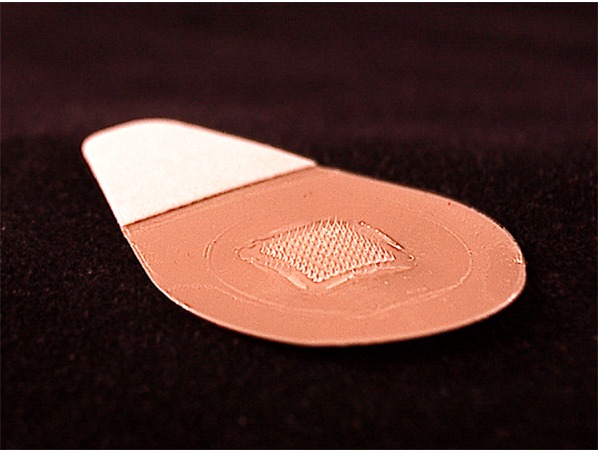
Microneedle patch (courtesy of John Toon, Georgia Institute of Technology)

Microneedle patches (MNPs) are micron-scale solid conical structures made of dissolvable excipients on a patch backing that deliver vaccine antigens (as well as drugs) across the stratum corneum into the epidermis and dermis (Figure 1). The MNP is applied by simply pressing it against the skin. After few minutes, the microneedles dissolve releasing the vaccine (or drug) and the patch is subsequently removed leaving minimal to no sharp waste.

A recent first-in-humans clinical trial of single dissolvable MNPs for seasonal influenza vaccine showed that this novel vaccine delivery method is safe, immunogenic and preferred over traditional needle and syringe in a healthy adult population (18-49 years of age) [4]. There is no reason to believe that the safety profile of influenza vaccine delivered by MNPs is different in the immunocompromised host. In addition, MNPs have the potential of improving immunogenicity in high risk populations by targeting the skin, an immunologically rich site where Langerhans cells enhance antigen presentation to the downstream adaptive immune system. In animal studies, influenza vaccine delivered through MNPs resulted in an increased breadth of immunity, longer duration of protection and potential for dose sparing [5]. The intradermal administration of a lower dose of 9 micrograms instead of the regular 15 micrograms for each of the influenza vaccine strains is already FDA approved for prevention of influenza in adults between the ages of 18 and 64 years of age [6]. The use of intradermal hepatitis B vaccination in immunocompromised patients was shown to be an effective immunization strategy able to overcome the reduced immunogenicity of traditional vaccine delivery methods [7].

Also, nosocomial influenza infections are common on oncology wards emphasizing the importance of mandatory healthcare worker vaccination and the importance of immunization of family and close contacts. IDSA recommends that all close contacts (≥ 6 months of age) of immunocompromised hosts be vaccinated with IIV. However in the US, and despite the current universal recommendation, seasonal influenza vaccination coverage in adults is only 43% [8]. Live attenuated influenza vaccine (LAIV) could be given to healthy non-pregnant close contacts aged 2-49 years, however for the past 2 influenza seasons, the CDC’s Advisory Committee on Immunization Practices (ACIP) recommended against the use of LAIV due to poor efficacy notably in children.

Therefore, MNPs provide an attractive alternative to traditional intramuscular injection of influenza vaccine by being less invasive, especially for needle phobic patients. In addition, MNPs have the potential for self-administration. In the recently published phase 1 study, 96% of participants (48/50) stated they experienced no pain during the IIV MNP administration, and 70% (33/47) preferred the MNPs over other vaccine delivery methods for IIV. In addition, 25 subjects without any healthcare background were able to successfully self-administer IIV using MNPs [4]. This is in contrast to other intradermal vaccine delivery or even needle free jet injectors requiring skilled healthcare personnel for appropriate administration. In addition in the phase 1 study, the safety and immunogenicity profiles of self-administered MNP group did not differ from the group who received the IIV through MNPs applied by a trained healthcare worker. Also, the vaccine in the MNPs is stable at room temperature for at least a year [4] with minimal to no sharps waste allowing the potential for convenient in home vaccination.

MNPs can also be used with vaccine immunogens other than the seasonal influenza vaccine and many have been successfully tested by the MNP developer, Dr. Mark Prausnitz and his team from the Georgia Institute of Technology (personal communication).

It is crucial to have a better understanding of the immunological mechanisms leading to the protective immune response induced by MNPs in the phase 1 trial. More clinical trials are needed to confirm the early results in larger groups and especially in immunocompromised hosts including patients with malignancies. If the patch is shown to have better acceptability and better immunogenicity in cancer patients, it could increase influenza vaccine uptake and protection against flu morbidity and mortality in this patient population.
